# The Histidine-Rich Calcium Binding Protein in Regulation of Cardiac Rhythmicity

**DOI:** 10.3389/fphys.2018.01379

**Published:** 2018-09-27

**Authors:** Demetrios A. Arvanitis, Elizabeth Vafiadaki, Daniel M. Johnson, Evangelia G. Kranias, Despina Sanoudou

**Affiliations:** ^1^Molecular Biology Division, Biomedical Research Foundation, Academy of Athens, Athens, Greece; ^2^Department of Cardiothoracic Surgery, Cardiovascular Research Institute Maastricht, Maastricht University, Maastricht, Netherlands; ^3^Department of Pharmacology and Systems Physiology, University of Cincinnati College of Medicine, Cincinnati, OH, United States; ^4^Clinical Genomics and Pharmacogenomics Unit, 4th Department of Internal Medicine, Attikon Hospital, Medical School, National and Kapodistrian University of Athens, Athens, Greece

**Keywords:** calcium, homeostasis, contractility, ion channels, arrhythmia

## Abstract

Sudden unexpected cardiac death (SCD) accounts for up to half of all-cause mortality of heart failure patients. Standardized cardiology tools such as electrocardiography, cardiac imaging, electrophysiological and serum biomarkers cannot accurately predict which patients are at risk of life-threatening arrhythmic episodes. Recently, a common variant of the histidine-rich calcium binding protein (HRC), the Ser96Ala, was identified as a potent biomarker of malignant arrhythmia triggering in these patients. HRC has been shown to be involved in the regulation of cardiac sarcoplasmic reticulum (SR) Ca^2+^ cycling, by binding and storing Ca^2+^ in the SR, as well as interacting with the SR Ca^2+^ uptake and release complexes. The underlying mechanisms, elucidated by studies at the molecular, biochemical, cellular and intact animal levels, indicate that transversion of Ser96 to Ala results in abolishment of an HRC phosphorylation site by Fam20C kinase and dysregulation of SR Ca^2+^ cycling. This is mediated through aberrant SR Ca^2+^ release by the ryanodine receptor (RyR2) quaternary complex, due to the impaired HRC/triadin interaction, and depressed SR Ca^2+^ uptake by the sarco/endoplasmic reticulum Ca^2+^ ATPase (SERCA2) pump, due to the impaired HRC/SERCA2 interaction. Pharmacological intervention with KN-93, an inhibitor of Ca^2+^/calmodulin-dependent protein kinase II (CaMKII), in the HRC Ser96Ala mouse model, reduced the occurrence of malignant cardiac arrhythmias. Herein, we summarize the current evidence on the pivotal role of HRC in the regulation of cardiac rhythmicity and the importance of HRC Ser96Ala as a genetic modifier for arrhythmias in the setting of heart failure.

## Introduction

Arrhythmias are potentially life-threatening electrical manifestations of the heart, which are difficult to predict and treat. The underlying molecular and cellular mechanisms leading to arrhythmia triggering are only partly known. Previously, Na^+^ and K^+^ ion channels, which are known to modulate depolarization and repolarization, had been a focus of studies on arrhythmia pathogenesis (Remme and Bezzina, 2010; Schmitt et al., 2014). However, many cases cannot be explained by impairments in these two types of channels, suggesting that additional genes, such as calcium (Ca^2+^) homeostasis regulators, are implicated in cardiac arrhythmias (London, 2013). Indeed, abnormal Ca^2+^ handling by the sarcoplasmic reticulum (SR) has emerged as a significant contributor to arrhythmogenesis. This is based on accumulating evidence on the crucial role of SR Ca^2+^ homeostasis in cardiac contraction, the direct association of human mutations in SR Ca^2+^ release components with arrhythmias, as well as alterations in the expression levels and activity of key Ca^2+^ handling proteins in human heart failure (Antoons and Sipido, 2008; Pritchard and Kranias, 2009; Lompre et al., 2010; Landstrom et al., 2017). One of these proteins, namely histidine-rich calcium binding protein (HRC) has attracted considerable attention over the past decade, since the identification of the HRC Ser96Ala gene variant was associated with increased risk for malignant arrhythmias and sudden cardiac death (SCD) in dilated cardiomyopathy patients (Arvanitis et al., 2008, 2011; Pritchard and Kranias, 2009). Deciphering the fine mechanisms of HRC contribution to cardiac physiology and pathophysiology could hold answers for the treatment of arrhythmias.

Herein, we summarize the major evidence on the crucial role of HRC in the regulation of cardiac rhythmicity through modulation of SR Ca^2+^ homeostasis, the clinical and molecular defects associated with the HRC genetic variant Ser96Ala, as well as potential therapeutic approaches targeting the underlying molecular defects in Ser96Ala carriers to prevent arrhythmia triggering.

## The Histidine-Rich Calcium Binding Protein

Histidine-rich calcium binding protein is a 170 kDa protein that is primarily expressed in striated muscles and arteriolar smooth muscle cells (Hofmann et al., 1989a,b; Pathak et al., 1992; Anderson et al., 2004). It is a highly charged molecule, with over 30% of the protein composed of acidic residues and 13% of histidine (Hofmann et al., 1989b). Based on its deduced amino acid sequence, HRC contains the following structural features: (1) an amino-terminal 27-residue signal sequence that is believed to target the protein to the SR; (2) a highly repetitive region containing 9 nearly identical tandem repeats of 29 residues, each consisting of a histidine-rich sequence HRHRGH and a stretch of 10–11 acidic amino acids that is believed to have Ca^2+^ binding properties; (3) a 13-residue stretch of polyglutamic acid; and (4) a cluster of 14 closely spaced cysteine residues, with the repeating pattern of Cys-X-X-Cys suggestive of a heavy metal binding domain, at the carboxyl terminus (Hofmann et al., 1989b). Detailed biochemical analysis has established that HRC is a low-affinity, high capacity Ca^2+^ binding protein. Although there is no Ca^2+^ binding motif in HRC, it is presumed that the acidic repeats constitute its Ca^2+^ binding sites (Hofmann et al., 1989b). It is noteworthy that HRC contains more acidic clusters than the other major SR Ca^2+^ binding protein, calsequestrin (CSQ), indicating the high capacity of HRC for Ca^2+^ binding (Hofmann et al., 1989a; Picello et al., 1992). These structural features render HRC an important regulator of cardiomyocyte SR Ca^2+^ homeostasis, as revealed by in depth investigations at the *in vitro, ex vivo*, and *in vivo* levels.

## The Role of HRC as a Prognostic Marker of Arrhythmias

Heart failure is characterized by impaired cytosolic Ca^2+^ handling, a process predominantly governed by the SR, which leads to diastolic and systolic dysfunction of the cardiomyocyte and ultimately of the heart pump (Morgan et al., 1990). Early reports on the association of HRC with cardiac pathophysiology proposed a role in dystrophic cardiac calcification of inbred mouse strains (van den Broek et al., 1998). HRC has been previously associated with two human autosomal dominant cardiac diseases, the isolated cardiac conduction (de Meeus et al., 1995) and the familial heart block type 1 (Brink et al., 1995). Further studies in end-stage human heart failure reported a significant decrease in HRC protein levels (Fan et al., 2004), a finding also observed in experimental heart failure in mice (Fan et al., 2004) and dogs (Gupta et al., 2009). Collectively, these observations implicated HRC in cardiac pathophysiology and raised the need for further investigations on its precise role and value in the clinical setting.

In 2008 our group was the first to show a direct association of the HRC Ser96Ala polymorphism with malignant ventricular arrhythmias. Through the study of 123 idiopathic dilated cardiomyopathy (DCM) patients (referred to hospital for diagnosis or treatment of heart failure, including implantable cardioverter-defibrillator (ICD) implantation) and 96 healthy controls, the HRC genetic variant Ser96Ala was found to have a statistically significant association with malignant ventricular arrhythmias and SCD in the patients (Arvanitis et al., 2008). Specifically, idiopathic DCM patients harboring the Ala/Ala variant had a fourfold increased risk of SCD compared to homozygotes for the Ser allele. The HRC Ser96Ala polymorphism was an independent predictor of life-threatening ventricular arrhythmias controlled by age, sex, left ventricular ejection fraction, atrial fibrillation, left bundle branch block or medication. Interestingly, further genetic analysis showed that approximately 60% of the general population has at least one copy of Ser96Ala, however, only those individuals suffering from DCM and carrying this polymorphism appear to be susceptible to arrhythmogenesis. Consequently, the HRC Ser96Ala polymorphism has a significant prognostic value.

## Understanding the Role of HRC in Arrhythmias Through *in Vitro* and *in Vivo* Rodent Models

One of the first studies to explore the role of HRC in SR Ca^2+^ handling, employed rat cardiomyocytes overexpressing HRC, and demonstrated that increased HRC levels lead to a 30% increase in SR Ca^2+^, accompanied by a 40% decrease in SR Ca^2+^ release, ultimately resulting in depressed cardiomyocyte shortening and re-lengthening. The depressed contraction of the HRC-infected cardiomyocytes remained even after maximal isoproterenol stimulation. At the molecular level, HRC overexpresssion was accompanied by increase of the ryanodine receptor (RyR2) quaternary complex components junctin and triadin (Fan et al., 2004).

This study was followed by the generation of a transgenic mouse model overexpressing HRC (by 3-fold). Genetically modified mouse models, as well as their explanted hearts and isolated cardiomyocytes, have long been used to delineate the effects of various genes and mutations thereof on cardiac arrhythmogenesis. Although these models have a number of limitations, they remain one of the major tools available for in depth cellular and physiological studies at the *in vivo, ex vivo*, and *in vitro* levels (Davis et al., 2012; Huang, 2017).

In this setting, the increased cardiac HRC levels were associated with impaired SR Ca^2+^ uptake (by 35%) and attenuated cardiomyocyte Ca^2+^ transient decay (by nearly 40%). Possibly as a compensatory mechanism, there was a marked increase of the Na^+^-Ca^2+^ exchanger (NCX) and triadin protein levels in myocytes isolated from these mice. At the cellular level, however, neither NCX mediated Ca^2+^ extrusion nor RyR2 SR Ca^2+^ release was elevated. In fact, the depressed SR Ca^2+^ sequestration was associated with an attenuated rate of Ca^2+^ extrusion. Interestingly, as the mice aged, they presented with impaired cardiomyocyte Ca^2+^ cycling, leading to abnormal cardiac remodeling and congestive heart failure (Gregory et al., 2006).

Similarly, the implications of the lack of HRC were investigated at the *in vitro* and *in vivo* levels. Initially, HRC was knocked-down in neonatal rat ventricular cells and HL-1 cells, using *in vitro* siRNA technology. This led to increased activity of RyR2 and SERCA2 whilst SR Ca^2+^ load was unaffected. The *in vivo* adeno-associated virus (AAV)-mediated HRC knock-down in mice with or without transverse aortic constriction (TAC)-induced heart failure, led to decreased fractional shortening and increased cardiac fibrosis compared with control. At the molecular level these findings were associated with increased phosphorylation of RyR2, CaMKII, p38 MAPK, and phospholamban (PLN). Thus, down-regulation of HRC led to significant deterioration of cardiac function following TAC-induced heart failure (Park et al., 2012).

Following these studies, HRC-knockout (KO) mice were generated. Although morphologically and histologically they were normal, compared to wild-type, at the cellular level, the HRC-KO mouse cardiomyocytes exhibited significantly enhanced contractility, Ca^2+^ transients, and maximal SR Ca^2+^ uptake rates. They also presented with an increased number of spontaneous SR Ca^2+^ releases and delayed afterdepolarizations, perhaps driven by the increased number of Ca^2+^ sparks seen in KO mice compared to controls. Under stress conditions of 1 μmol/L isoproterenol and 5 Hz stimulation, the HRC-KO isolated cardiomyocytes were found to be five-fold more prone to after-contractions compared to wild-type mice. After TAC the HRC-KO mice developed severe cardiac hypertrophy, fibrosis, pulmonary edema and premature death compared to wild-types. Isolated cardiomyocytes from TAC treated HRC-KO hearts exhibited poor contractility and impaired Ca^2+^-cycling, possibly due to reduced SERCA2 expression. These data underline the importance of HRC in the normal physiology of SR Ca^2+^ handling, as well as in maintaining the integrity of normal cardiac performance (Park et al., 2013).

Since HRC Ser96Ala has been associated with malignant ventricular arrhythmias (Arvanitis et al., 2008), the cellular mechanisms of pathogenesis were investigated initially in isolated adult rat ventricular cardiomyocytes infected with adenoviruses encoding the human HRC (hHRC) Ser96 or Ala96. The hHRC Ala96 exacerbated the inhibitory effects of hHRC Ser96 on the amplitude of Ca^2+^ transients, the prolongation of Ca^2+^ decay, and the caffeine-induced SR Ca^2+^ release, indicating a higher SR Ca^2+^ load. hHRC Ala96 had a stronger suppressive effect on the maximal SR Ca^2+^ uptake rate compared to hHRC Ser96. Furthermore, hHRC Ala96 increased the frequency of spontaneous Ca^2+^ sparks, while the hHRC Ser96 reduced them compared to GFP expressing controls. Importantly, expression of hHRC Ala96 in cardiomyocytes from a rat model of post-myocardial infarction heart failure induced dramatic disturbances of rhythmic Ca^2+^ transients. These findings reproduce the arrhythmogenic propensity of the human HRC Ser96Ala variant carriers when their cardiac function is compromised in the setting of heart failure (Han et al., 2011).

A recent study was carried out on a ‘humanized’ mouse of HRC Ser96Ala, where the murine HRC gene was replaced by the human homolog, and therefore carried either the wild-type gene with serine at position 96, or the alanine variant. The hHRC Ala96 mice displayed poor contractility and Ca^2+^ cycling compared to hHRC Ser96, in the absence of any structural or histological abnormalities. The occurrence of Ca^2+^ waves was significantly higher but the SR Ca^2+^ load was lower in hHRC Ala96 compared to hHRC Ser96. At the molecular level these findings were attributed to the poor interaction of HRC Ala96 with triadin, and ultimately with RyR2 (through triadin). Indeed, the open probability of RyR2 was significantly increased in hHRC Ala96 cardiomyocytes. Under stress conditions of 1 μmol/L isoproterenol and 5 Hz stimulation, isolated cardiomyocytes from hHRC Ala96 hearts presented with 6-fold more aftercontractions and 3-fold more delayed afterdepolarizations when compared to hHRC Ser96 cardiomyocytes. RyR2 was found to be hyperphoshorylated by CaMKII at Ser2814. Importantly, treatment of the hHRC Ala96 derived cardiomyocytes with KN93, a specific inhibitor of CaMKII, reversed the increase of Ca^2+^ sparks and waves (Singh et al., 2013).

Although these studies provided important insights into the potential arrhythmogenic mechanisms of the Ser96Ala mutation, they had an inherent issue – a non-murine protein being produced in the mouse. This could result in interactions of the human HRC with mouse proteins that may not necessarily take place when exclusively murine HRC is present. We therefore pursued an alternative, and perhaps more physiologically relevant approach, where the mutation was directly introduced in the mouse gene, at the equivalent site. This led to the development of the HRC Ser81Ala mouse model, which recapitulated the human Ser96Ala mutation directly in the mouse HRC protein (Tzimas et al., 2017). The HRC Ser81Ala mice presented with increased mortality over a 12-month period, with 50% of the Ser81Ala mice dead by 10 months, compared to only 15% of the wild-type animals. Interestingly, no abnormalities were noted in the Ser81Ala mouse hearts in terms of cardiac structure and fibrosis, at least as far as 3–5 months of age, similar to data from the humanized Ser96Ala mouse. In addition, cardiac contraction, both *in vivo* and *ex vivo* (as measured by Langendorff preparation), was shown to be significantly decreased in 3–4-month-old Ser81Ala mice, when compared to the wild-types, whilst the Ser81Ala mice showed a slight decrease in intraventricular septum and left ventricular posterior wall thickness. Further age-related decreases in contractile function were similar between the two strains. Taken together, these data indicated that the increased mortality observed in HRC Ser81Ala mice was unlikely due to acute heart failure, and other factors should be considered (Tzimas et al., 2017).

Due to the fact that ventricular arrhythmias have been previously noted in the patient population with the Ser96Ala variant (Arvanitis et al., 2008), we hypothesized that SCD as a result of arrhythmias may also be the reason for the increased mortality in the Ser81Ala mice. We therefore performed a series of electrophysiological examinations on these mice *in vivo*. This work revealed an increased sensitivity of the Ser81Ala mice to an arrhythmogenic challenge (caffeine/epinephrine administration) when compared to the wild-type, with more than 50% of Ser81Ala mice going into ventricular tachycardia, compared to none of the wild-type animals. We extended these studies by carrying out experiments in isolated myocytes, where an increase in aftercontractions, calcium after-transients and delayed afterdepolarizations were observed in Ser81Ala derived cells, further supporting the hypothesis that the increase in mortality is due to arrhythmogenic events (Tzimas et al., 2017). Interestingly, contraction in myocytes from the Ser81Ala mouse was not increased, indicating that Ca^2+^ overload, *per se*, was not a driving force for the arrhythmogenic phenotype, as has been seen in a number of other models and digitalis-induced arrhythmias (Sipido, 2006; van den Hoogenhof et al., 2018).

Interestingly in heart failure, where arrhythmias may also ensue, it has been shown that SR Ca^2+^ load is not increased (Hasenfuss and Pieske, 2002). In this case arrhythmogenesis may be due to a variety of other factors, including increased activity of RyR2 or altered activity of NCX. For these reasons we went on to investigate if there was an increased spontaneous Ca^2+^ release in the Ser81Ala cardiomyocytes. Despite the fact we did not observe any alterations in Ca^2+^ spark properties, we did see an increase in total Ca^2+^ leak in myocytes isolated from the Ser81Ala mice, and an increase in Ca^2+^ wave speed. Furthermore, an increase in phosphorylation of RyR2 was noted at the CaMKII phosphorylation site (Ser-2814) under baseline conditions in Ser81Ala myocytes, whilst no differences were observed at the protein kinase A (PKA) site of RyR2, or in PLN phosphorylation. However, there were no alterations in the levels of the RyR2 protein, whilst an increase was seen in junctin and NCX, and a decrease in triadin. Additionally, increases in action potential (AP) duration were noted in the Ser81Ala myocytes, which went along with an increase in inward (Ca^2+^) current. Taking all of these data together, we hypothesized that CaMKII played a key role in the arrhythmogenic phenotype in the Ser81Ala mouse. At both the cellular and *in vivo* levels, inhibition of CaMKII with KN-93 was indeed able to reduce the number of arrhythmogenic events.

Despite the fact that further studies should be carried out in this model, including the potential dyadic structural remodeling in myocytes, we can summarize that the HRC Ser81Ala mutation causes arrhythmia via increased spontaneous Ca^2+^ leak and longer APs, and this is, at least in part due to CaMKII activity. This alteration of CaMKII activity could be driven by the alterations in the local Ca^2+^ concentration, although the exact mechanisms of CaMKII activation in this model remains to be elucidated. Taking all of these data into account, CaMKII inhibition could be a potential therapeutic target in patients with similar polymorphisms/mutations.

## HRC Binding Partners and Their Role in Mediating HRC Effects on Ca^2+^ Homeostasis and Arrhythmogenesis

**FIGURE 1:**
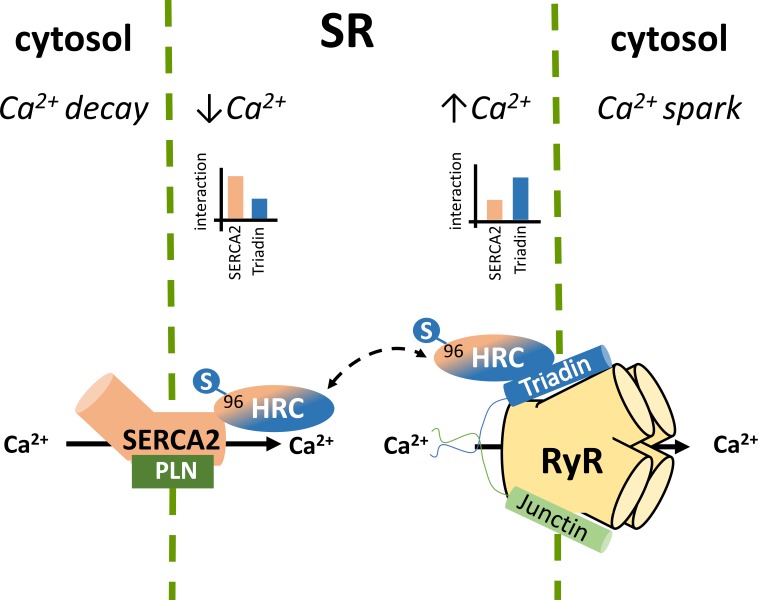
Histidine-rich calcium binding protein complex cellular interactions within the cardiomyocytes. Local SR Ca^2+^ changes alter the dynamics of HRC interactions with triadin and SERCA2.

The SR holds a pivotal role in Ca^2+^ compartmentalization within the cardiomyocyte. During each cycle of cardiomyocyte activation Ca^2+^ fluxes rapidly back and forth between the cytosol and the sarcoplasmic reticulum. The SR Ca^2+^ release occurs through the opening of the gigantic RyR2 multiprotein complex under the regulation of triadin and junctin (Gambardella et al., 2018; Santulli et al., 2018; Slabaugh et al., 2018), while the energetic transport of cytosolic Ca^2+^ to the SR is achieved by SERCA2 under the regulation of PLN (Vafiadaki et al., 2009; Wang et al., 2011; Kranias and Hajjar, 2012). The HRC protein interacts with both systems mediating a fine cross talk between cytosolic Ca^2+^- spark and decay (**Figure 1**; Arvanitis et al., 2007). HRC has been previously shown to be localized to the SR lumen in cardiac and skeletal muscles (Hofmann et al., 1989a; Shoshan-Barmatz et al., 1996; Suk et al., 1999) and based on its structural similarities with CSQ, it was initially suggested to be functionally associated with SR Ca^2+^ release (Hofmann et al., 1989a; Damiani and Margreth, 1991). This was supported by several studies, clearly demonstrating the direct interaction of HRC with triadin, an integral SR membrane protein forming a quaternary complex with the RyR2, CSQ, and junctin (Kim et al., 1990; Sacchetto et al., 1999, 2001; Lee et al., 2001). HRC binds to triadin in a Ca^2+^-dependent manner, with increases in Ca^2+^ concentration leading to enhanced interaction between the two proteins (Sacchetto et al., 2001; Arvanitis et al., 2007; Rani et al., 2016). Multiple domains of HRC, including the C-terminal cysteine-rich domain (Sacchetto et al., 1999, 2001; Arvanitis et al., 2007) and the histidine-rich and acidic repeats (Lee et al., 2001), have been shown to interact with triadin. Interestingly, HRC interacts with triadin’s luminal KEKE motif, a region which is also implicated in CSQ and RyR2 binding (Lee et al., 2001; Rani et al., 2016). Given this overlap on triadin’s minimal binding region, a biochemical study recently examined the potential competition between CSQ, HRC and RyR2 for triadin binding. Using increasing concentrations of RyR2 or CSQ peptides in immunoprecipitation assays, HRC was demonstrated to compete with RyR2 and CSQ for binding to triadin (Rani et al., 2016). These findings, indicate the regulatory nature of these protein associations that are tightly controlled by Ca^2+^ concentration toward effective regulation of RyR2 function and SR Ca^2+^ release. The possibility that HRC can bind to RyR2 independently of triadin, was suggested by a study utilizing the HEK293 cell culture system which lacks endogenous triadin protein (Zhang et al., 2014). However, as these findings were obtained solely from microscopy-based assays, and no further evidence has emerged since then to support this notion, additional work is required to establish this association. Overall, based on its association with triadin, HRC is believed to modulate RyR2 function and SR Ca^2+^ release by conferring refractoriness to SR Ca^2+^ release and exerting a presumably beneficial effect on SR Ca^2+^ reloading at intermediate Ca^2+^ concentrations (Rani et al., 2016).

In addition to triadin, we have demonstrated that HRC directly interacts with SERCA2a, the key enzyme mediating SR Ca^2+^ uptake (Arvanitis et al., 2007). Similarly to the HRC-triadin, the HRC-SERCA2 interaction is also Ca^2+^-dependent, but with maximal binding occurring at low Ca^2+^ concentration, and a diminishing HRC-SERCA2 association with increasing Ca^2+^ concentrations (Arvanitis et al., 2007). The protein domains involved in this interaction include the amino-terminal fragment of SERCA2 that projects into the SR lumen (amino acid residues 74–90) and the second histidine- and glutamic acid-rich domain of HRC (amino acids 320–460) (Arvanitis et al., 2007). Interestingly, the HRC domain binding to triadin is distinct, and involves its cysteine-rich domain (amino acids 609–699) at the carboxyl terminal fragment (Sacchetto et al., 2001; Arvanitis et al., 2007). The choice of HRC binding partner, which is tightly controlled by Ca^2+^ concentrations, determines the HRC effect on SR Ca^2+^ cycling. At low SR Ca^2+^ load HRC interacts with SERCA2 and inhibits its activity. However, upon increases in SR Ca^2+^ concentration, HRC dissociates from SERCA2, exhibits enhanced binding to triadin, and modulates SR Ca^2+^ release (Arvanitis et al., 2011). Through this dual association with SERCA2 and triadin, HRC is implicated in both SR Ca^2+^ uptake and Ca^2+^ release, with defects in both processes observed upon HRC overexpression or ablation (Gregory et al., 2006; Park et al., 2013). HRC may therefore represent a key mediator of the fine cross-talk between these two processes in cardiomyocyte contraction and relaxation. Any factors modulating HRC, such as post-translational modifications (see section below) or gene sequence variations (e.g., the genetic variant Ser96Ala), could affect these protein associations with direct implications in SR Ca^2+^ cycling and arrhythmogenesis.

**FIGURE 2:**
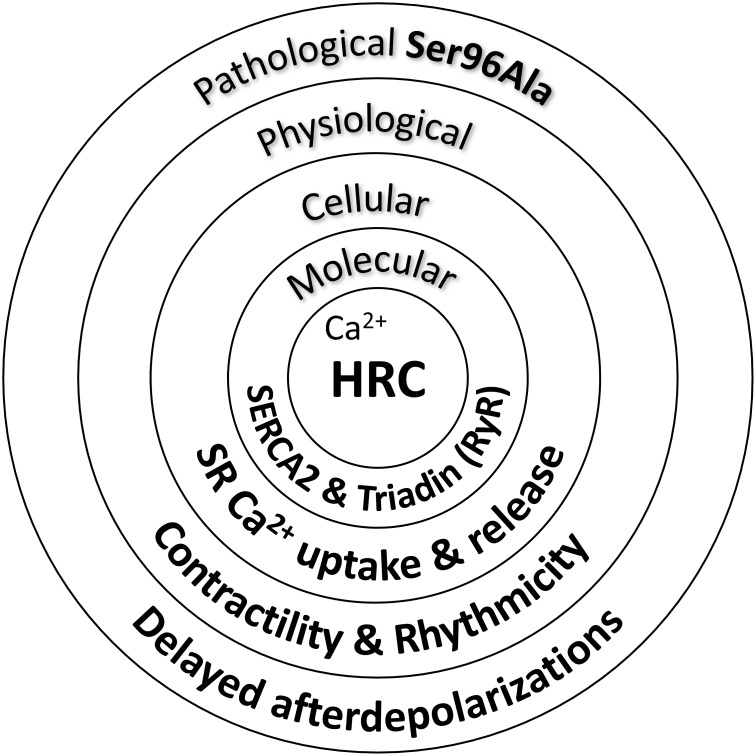
Histidine-rich calcium binding protein functional significance in cardiac physiology and pathophysiology.

Indeed, acute overexpression of the HRC Ser96Ala human variant in cell culture systems significantly increased the binding of HRC Ser96Ala to SERCA2, an effect that was associated with enhanced inhibition of SERCA2 activity and thus reduced maximal SR Ca^2+^ uptake rate (Han et al., 2011). Importantly, HRC Ser96Ala increased the frequency of spontaneous Ca^2+^ sparks, suggesting enhanced RyR2 activity, and leading to increased uncontrolled SR Ca^2+^ release (Han et al., 2011). Similarly, as mentioned above, cardiomyocytes from the humanized HRC Ser96Ala mouse model exhibited increased RyR2 activity, enhanced propensity for spontaneous SR Ca^2+^ release and arrhythmias (Singh et al., 2013). Collectively, these studies have clearly demonstrated the functional significance of HRC interactions with SERCA2 and triadin toward effective regulation of SR Ca^2+^ uptake and release (**Figure 2**). Defects in HRC function due to genetic mutations lead to aberrant SR Ca^2+^ cycling and enhanced propensity toward arrhythmia triggering.

## HRC Post-Translational Modification

Initial reports on the HRC gene and protein functions described the phosphorylation of HRC by multiple kinases, including PKA and CaMKII (Damiani and Margreth, 1991; Damiani et al., 1995), as well as casein kinase II (CKII), an endogenous kinase which associates with SR membranes (Orr and Shoshan-Barmatz, 1996; Shoshan-Barmatz et al., 1996). Although PKA and CaMKII can phosphorylate HRC *in vitro*, these phosphorylations may not occur *in vivo* since HRC is a luminal protein and is not accessible to these cytoplasmic kinases (Hofmann et al., 1989a; Suk et al., 1999). It has been postulated that phosphorylation of HRC by CKII decreased the affinity of RyR2 for Ca^2+^, although it was not clear whether this phosphorylation mediates a direct effect on RyR2 activity (Orr and Shoshan-Barmatz, 1996; Shoshan-Barmatz et al., 1996).

The discovery of the HRC Ser96Ala variant association with life-threatening ventricular arrhythmias in the setting of dilated cardiomyopathy, led to the hypothesis of abolishment of a potential phosphorylation site on HRC, with direct implication in downstream molecular and cellular processes. Indeed, bioinformatical analysis showed that HRC Ser96, an evolutionary conservative amino-acid position, could be phosphorylated (Arvanitis et al., 2008). *In vivo* phosphoproteomic studies of healthy human skeletal muscle found that HRC is phosphorylated at six serine amino acid residues, namely Ser119, Ser431, Ser563, Ser567, Ser157/Ser159, and Ser170/Ser171 (Hojlund et al., 2009). The authors predicted, using bioinformatical analysis, that the majority of these sites, (Ser119, Ser170 or Ser171, Ser431, and Ser567), could be phosphorylated by the luminally located CKII, while Ser157 or Ser159 may be phosphorylated by protein kinase B (PKB), and Ser563 by the mitogen-activated protein kinase 8 (MAPK8) or the glycogen synthase kinase 3 beta (GSK3B). Another study, which focused on *in vitro* dephosphorylation of cardiac proteins, demonstrated that HRC is phosphorylated at five positions (Ser104, Ser129, Ser249, Ser324, and Ser466) in wild-type hearts and can be dephosphorylated *in vitro* by alkaline phosphatase (Husberg et al., 2012). Although these studies were valuable for achieving a deeper understanding on post-translational modifications of wild-type HRC in healthy human skeletal and cardiac muscles, the status of HRC Ser96 remained unaddressed.

**FIGURE 3:**
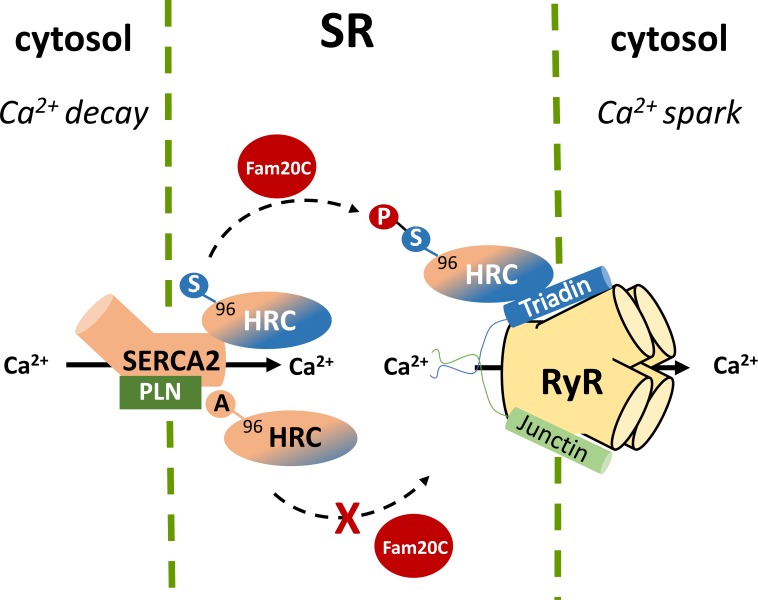
Histidine-rich calcium binding protein phosphorylation by Fam20C at Ser96 affects the affinity of HRC interactions with SERCA2 and triadin while the Ala96 variant remains unaffected.

Recently, a new kinase, family with sequence similarity 20C (Fam20C) that phosphorylates S-x-E/pS motifs on proteins was found to be responsible for the majority of the extracellular phosphoproteome (Tagliabracci et al., 2015). Located within the ER and the Golgi apparatus, Fam20C phosphorylates a multitude of proteins including HRC Ser96, Ser119, Ser145, Ser358, Ser409, Ser431, Ser494, and Ser567. These findings not only identify Fam20C as the predominant kinase involved in the protein secretory pathway of cells but also suggest that Fam20C is a potential pharmacological target, affecting a multiple of cellular processes ranging from cell adhesion and migration to treatment against HRC driven life-threatening ventricular arrhythmias. Further proteomic and biochemical investigations under native or post-phosphatase treated whole-cardiac homogenates, clearly demonstrated that Fam20C is the kinase for HRC *in vivo* (Tagliabracci et al., 2015). These findings were supported by cell culture studies of co-expression of HRC and Fam20C in rat cardiomyoblasts, and expression of HRC in human osteosarcoma cells with CRISPR/Cas9 knockout of Fam20C. The importance of HRC phosphorylation at position 96 was demonstrated when HRC KO mouse heart derived primary cardiomyocytes, which exhibit increased arrhythmogenicity, were infected with pseudo-phosphorylated (HRC-Asp96) encoding adenoviruses, which alleviated the arrhythmias. In addition, the Fam20C-phosphorylated HRC showed a stronger interaction with triadin and a weaker interaction with SERCA2 by co-immunoprecipitation studies, while the unphosphorylated HRC showed a stronger interaction with SERCA2 (**Figure 3**). Collectively, these data suggest that Fam20C is, at least in part, responsible for HRC phosphorylation, and that the variant Ser96Ala changes the affinity of HRC interactions with triadin and SERCA2 (Pollak et al., 2017). Further experiments with specific inhibitors of Fam20 will be required to clarify the role of Fam20-phosphorylation in arrhythmia generation.

## Conclusion

Studies on HRC function over the past decade, using *in vitro* and *in vivo* approaches at the molecular, cellular, tissue, organ, and whole organism levels have demonstrated its importance in cardiomyocyte physiology and pathophysiology. HRC mediates a cross-talk between SR Ca^2+^ uptake and release, regulating both systems and acting as a Ca^2+^ sensor. Importantly, the function of HRC is regulated by its phosphorylation at Ser96 and human carriers with the Ser96Ala variant are predisposed to cardiac arrhythmias under stress conditions. Thus this variant may serve as a biomarker for the identification of cardiovascular disease patients at risk for SCD.

## Author Contributions

All authors contributed in manuscript preparation and approved the final version of the manuscript.

## Conflict of Interest Statement

The authors declare that the research was conducted in the absence of any commercial or financial relationships that could be construed as a potential conflict of interest.
